# From Their Point of View: Identifying Socio-Behavioral Profiles of Primary School Pupils Based on Peer Group Perception

**DOI:** 10.3389/fpsyg.2018.01421

**Published:** 2018-08-07

**Authors:** Laura E. Prino, Tiziana Pasta, Claudio Longobardi, Davide Marengo, Michele Settanni

**Affiliations:** Department of Psychology, University of Turin, Turin, Italy

**Keywords:** cluster analysis, social skills, social development, student–teacher relationship, peer nomination, child behavior, elementary school

## Abstract

Our study adopted a person-based approach with the aim to identify socio-behavioral profiles of primary school students based on peer group perception. The study involved 109 classes and their teachers, from the first three grades of elementary school. The final student sample consisted of 424 children, aged 6–9 years (*M* = 94.9 months; *SD* = 9.7), of whom 58.3% were male. We used peer-group nomination to investigate the aspects that are linked to peer group acceptance and perception of classroom behaviors, with reference to academic and relational criteria. We identified and defined six clusters. We validated these clusters by taking into consideration the children’s academic performances and the teacher’s perceptions of their relationship with the single students. The identified clusters were related to both of these aspects, and they show predictive value when referring to children’s behaviors as evaluated by their teachers. Implications for theory and educational policies are discussed.

## Introduction

### The Importance of Relationships in Scholastic Settings and Their Effects

Many authors argue that schools represent, after families, one of the most important settings for children’s acquisition of new competences and their development of cognitive, social, and emotional skills (Collins et al., 2017; Longobardi et al., 2017; McLeod et al., 2017).

Considering the amount of time that children spend at school with their peers, the peer group is an important context in which children have the possibility of testing their social competences and learning new ways of interacting (Acquah et al., 2014; Sentse et al., 2017; Longobardi et al., 2018b). The developmental advantages that derive from the positive relationships experienced with peers, are to be compared with the negative effects that stem from the rejection that children may sometimes experience at the hands of their peer group. In fact, not all children manage to establish and maintain positive relationships with their peers. Research shows that poor quality peer relationships are correlated with present and future difficulties in adjustment, antisocial behavior (e.g., delinquency, dropping out of school), poor academic performance and mental health problems (Hymel et al., 2002; Sentse et al., 2017). For these reasons, relationships with classmates constitute, by themselves, crucial support for the child’s emotional, social, cognitive, and behavioral development (Kupersmidt and Coie, 1990; Longobardi et al., 2018a).

Particularly, the relationships formed during the first years of scholarization are more relevant because they constitute an important predictive factor for the scholastic adjustment and integration that will follow during the subsequent years (Longobardi et al., 2016a,b). In fact, various studies have recognized the correlation between the quality of peer group relationships and children’s social behavior, as well as its long-term effects (Acquah et al., 2014).

A number of studies have recognized that prosociality, being a social competence, increases the possibility of having a positive developmental trajectory, while playing a crucial role in influencing children’s scholastic proficiency and their academic success, both present and future (Rubin et al., 1998; Eisenberg et al., 2006; Junttila et al., 2006).

However, children that have been cast out from the group frequently display aggressive and disruptive behaviors (e.g., arguing, breaking things, hitting, threatening, or insulting someone) (Bukowski, 2003). In fact, isolation from the peer group can contribute to maintaining aggressive behavioral responses that are enacted as a reaction to the exclusion from the group (Poulin and Boivin, 2000).

Furthermore, various studies have highlighted how children who present behaviors such as shyness or social withdrawal could be at risk of peer group rejection, and how they could manifest a wide range of sociorelational difficulties (Rubin et al., 2011).

Also for what concerns aggressive behavior, many studies have emphasized its stability since infancy and its predictive value, not just for peer group rejection, but also for future problematic behaviors, such as academic failure and dropout, psychopathology, behaviors that are linked to low self-control, or also overtly delinquent behaviors such as theft and violent aggression (Olweus, 1981).

The study of behavioral characteristics, social relationships, and their effects during the first years of primary school is a crucial research topic if we wish to improve our understanding of the factors that can play a role in modifying developmental trajectories (Sawyer et al., 1997; Bouchard et al., 2015).

Many studies on social behaviors have investigated gender differences: the literature has highlighted differences with reference both to problematic behaviors (e.g., hyperactivity and attention deficit, relational difficulties with peers, conduct problems, etc.), more common in males, and to prosocial behaviors, more common in females (Pianta et al., 1995; Rose and Rudolph, 2006; Tobia et al., 2011; Quaglia et al., 2013). Traditionally, studies have also reported higher levels of overt aggression in boys (Buss, 1961; Coie and Dodge, 1998), and of relational or social forms of aggression in girls (Feshbach and Feshbach, 1969; Lagerspetz et al., 1988). More recently, a number of scholars have pointed out a few issues concerning the interpretation of these results, on the one hand questioning the heterogeneity of the measurement methods that had been employed in the studies and, on the other hand, stressing the importance of interpreting gender differences in the light of their social and cultural significance (Eisenberg et al., 2006; Junttila et al., 2006; Neal and Cappella, 2012).

According to the Socio-ecological Perspective (Bronfenbrenner, 1979; Pellegrini, 2008) in fact, it is essential to know the meaning that behaviors acquire inside an interactive and relational context, and in the light of the processes through which they occur. For example, by considering aggressive behaviors, traditionally a large number of studies have argued the connection between peer-group rejection, aggressive behavior and social incompetence (Coie et al., 1991; Farmer et al., 2003; Rodkin and Roisman, 2010). Instead, more recent contributions, as Resource Control Theory (Hawley, 2002, 2007, 2008), have highlighted that aggressiveness is not necessarily a maladaptive trait, but it can be used efficiently to reach one’s goals by acquiring resources while maintaining positive relationships inside the peer group. Since the mid-1990s, in fact, studies have recognized the presence of a subgroup of children who present aggressive behaviors, combined with good social skills, and who are considered socially appealing (Cairns and Cairns, 1994; Rodkin et al., 2000; Roseth et al., 2007).

### Studying Children’s Behaviors in the Classroom

Both variable-centered, and person-centered methods have been applied to study children’s behavioral characteristics.

Both approaches have brought, and continue bringing, relevant contributions to our knowledge base, and although said contributions may be different, they are often complementary (Bergman and Magnusson, 1997; Hawley et al., 2007).

In the studies on children’s social behaviors the variable-centered approach was employed mostly for investigating the linear relation between social acceptance and aggressive behaviors (Cillessen and Mayeux, 2004). Instead, the person-centered approach was used to facilitate the identification of homogeneous subgroups of children that share common patterns of the variables under study (Kruskal and Wish, 2000; Rodkin et al., 2000; Hawley et al., 2007).

A group of person-centered studies considers the evaluation of children’s behavior from their peers’ point of view, taking into account their social status (Bagwell et al., 2000; Trilivas and Chinimenti, 2000) either to study the relation between social acceptance and aggression (Rodkin et al., 2000; Hawley et al., 2007; Estell et al., 2008; Marengo et al., 2018), or to investigate different behaviors in classrooms, such as prevaricating and victimizing dynamics (Acquah et al., 2014), sometimes studying male and female behavioral typologies separately (Rodkin et al., 2000; Estell et al., 2008).

In the majority of these studies, the profile that proved to be the most numerous was composed by students that were defined as “average”; they showed “normal” behaviors and a low presence of problematic situations (Rodkin et al., 2000; Estell et al., 2008; Bulotsky-Shearer et al., 2010, 2012; Acquah et al., 2014). There was also a group of “troubled” subjects that presented relevant behavioral difficulties, and were unpopular, rejected or at high risk of isolation (Bagwell et al., 2000; Rodkin et al., 2000; Estell et al., 2008; Bulotsky-Shearer et al., 2010; Robertson et al., 2010; Acquah et al., 2014). Conversely, there was also a group of subjects (sometimes labeled as “models”) that presented high scores in adaptive or prosocial behaviors, combined with the absence of problematic behaviors and a general popularity among their peers, (Huberty et al., 1997; Rodkin et al., 2000; Estell et al., 2008). In closing, a number of studies (Bagwell et al., 2000; Rodkin et al., 2000; Roseth et al., 2007; Estell et al., 2008; Robertson et al., 2010; Acquah et al., 2014) has highlighted the presence of a group of aggressive subjects that hold central positions inside the social structure (in one example labeled “tough” by Estell et al., 2008).

Independently from the methodological approaches employed by the various studies, the information gathered from teachers, parents or external evaluators has often been used as the basis for studying classroom social dynamics. However, the limitation of basing the research on data that is unique and external to the relational dynamics of the peer group has been pointed out (Pepler and Craig, 1998; Pellegrini and Bartini, 2000). Peers, in fact, have the possibility of observing a wider range of behaviors that are enacted by children in different contexts, and also in those moments in which adult supervision is not present (Craig and Pepler, 1995; Craig et al., 2000). When peer assessment is employed, researchers can rely on multiple informers instead of single ones (whose judgment can be influenced by personal expectations and experiences); in this manner, both the reliability and validity of the evaluations of the single child’s social behavior are increased (Pepler and Craig, 1998). Literature on peer assessment distinguishes between sociometric popularity (Coie et al., 1982) and perceived popularity (Parkhurst and Hopmeyer, 1998). The former is a measure of social preference, while the latter is a measure of social visibility (Cillessen and Mayeux, 2004). Traditionally, sociometric nomination measures divide children into five sociometric status categories: popular, rejected, average, controversial, and neglected (Coie et al., 1982). Sociometric popularity corresponds to an elevated number of liked nominations, and it is obtained by calculating the nominations of most-liked and least-liked peers. Perceived popularity, instead, is assessed by asking peers directly to point out which are the “popular” children in their class. The former is a measure of how much children are appreciated by their peers, while the latter is an indicator of the impact and social reputation of a single child (Cillessen and Mayeux, 2004). The literature has highlighted that the two constructs are only partially overlapping (Parkhurst and Hopmeyer, 1998; LaFontana and Cillessen, 1999), and that they are linked differently to personal and social characteristics (Rodkin et al., 2006; Acquah et al., 2014). Sociometrically popular children are considered as being reliable, sociable, kind, and cooperative. Said characteristics are also present among perceived popular children, but, in this case, they can also be combined with characteristics such as being dominant, arrogant, and aggressive, both in a physical and in a relational manner (LaFontana and Cillessen, 2002).

### Study Aims

On the whole past literature has focused on the development of students classifications based either on external evaluators’ (mainly teachers or parents) view or on popularity or social preference among peers. We aim at testing whether a typology of students can be developed based both on popularity and on how peers perceive schoolmates’ behaviors. The novelty of this approach is that only peer perceptions of how other students behave are considered in the development of the typology. Hence, our main goal is to expand previous research by identifying different socio-behavioral profiles among primary school students, based on peer-group perception. In order to achieve this goal, we will employ measures obtained solely through peer assessment, and relative to a wide range of children’s behaviors. For the purpose of evaluating the validity of the typology of behavioral patterns that will emerge from our study, we will analyze whether there is a correspondence between the emerging clusters and the different dimensions of their relationship with the teacher, as evaluated by the teacher themselves.

As a further validation for the typology of behavioral profiles, we will study the differences between the various clusters, with reference to the dimensions of gender and academic achievement.

As a final aim, in order to evaluate the usefulness of the typology in predicting actual children behavior in the school context, we will analyze the relationships between cluster membership and behaviors as evaluated by teachers.

## Materials and Methods

### Participants

The study was based in a Region of Northwestern Italy and it involved classrooms from the first three grades of elementary school. Twenty-five schools took part in the study. Six classrooms were randomly selected for each school (two for each grade). In each of the selected classrooms, we asked both the students and the teacher spending the highest amount of hours per week with the class to participate in the research. Eventually, 109 (out of 125) teachers (94% females) and 1940 students (44% females) agreed to participate. The total student sample was asked to take part into the peer nomination procedure. After that, for each classroom, four students (about 20% of the class students) were randomly selected and their teachers were asked to fill out questionnaires about each of them). Final sample consisted of the students who were evaluated by their teachers: They were 424 children, aged 6–9 years (*M* = 94.91 months; *SD* = 9.69), of whom 58.3% were male. In particular, 17% of the sample belonged to the first grade, 37% to the second grade, and 46% to the third grade.

### Instruments

#### Peer Nomination

To investigate the social characteristics of students, we used the peer nomination technique (Coie and Dodge, 1983; Newcomb and Bukowski, 1983; Salmivalli and Isaacs, 2005), which we applied individually to every participant. The items were designed to measure aspects referring to both social preference (items a and b) and perception of social behaviors (items c to h).

The children were asked to name one or more members of their classroom who best fit the descriptions for eight items. The participants could not name themselves and they could name the same classmate in more than one question. The eight descriptions were: (a) *Playing*. “Which children of your class do you like to play with the most?” (b) *Doing classwork*. “Who are the children you like to work with/solve problems with the most in your class?” (c) *Disturbing*. “Some children do strange things and make plenty of noise, disturbing others who are trying to work. Which children are like that in your class?” (d) *Helping*. “Some children like to help others and will gladly lend their things. Which children are like that in your class?” (e) *Arguing.* “Some children argue with their classmates, say bad things to them or hit them. Which children are like that in your class?” (f) *Being Isolated.* “Some children are left alone; classmates never ask them to do things together and others say bad things behind their backs. Does this happen to children in your class?” (g) *Getting along well with the teachers*. “Some children get along well with their teachers, they talk with them easily and even teachers like to spend time with them as well. Which children are like that in your class?” (h) *Getting laughed at.* “Some children get laughed at, provoked or hit by their classmates. Does this happen to children in your class?”

We used these eight items to investigate the aspects linked to popularity and perception of prosocial (items a, b, d and g), antisocial (c and e), and asocial behaviors (f and h).

We calculated the percentage of the nominations received by each participant in all questions, and divided these by the total number of classmates minus one, multiplying the obtained result by 100. The mean classroom percentage of students participating in the peer-nomination procedures was at least 75%. Peer nomination percentages were calculated for all class participants, but given our research design, only percentages referring to the students who were evaluated also by teachers (*N* = 424) were included in the subsequent analyses.

#### Student–Teacher Relationship Scale

To investigate the quality of the teacher’s representation of his or her relationship with specific pupils, we used the Student–Teacher Relationship Scale (STRS, Pianta, 2001) in its 22-item, Italian version (Fraire et al., 2013; Settanni et al., 2015). Every item was evaluated by the teacher on a five-point Likert scale (ranging from Not Applicable to Completely Applicable). The STRS investigates three dimensions of a relationship: Conflict, Closeness, and Dependency. Scales reliability was computed using Cronbach’s alpha, and it ranged between 0.75 (Dependency) and 0.84 (Closeness).

#### Strength and Difficulties Questionnaire

To investigate the behavioral characteristics of the children, we asked each teacher to fill out the Strength and Difficulties Questionnaire (SDQ, Goodman, 1997; Tobia et al., 2011), which is composed of 25 items that refer to the positive or negative traits of the child’s behavior in class. The instrument is divided into five subscales: Emotional Symptoms, Behavioral Problems, Hyperactivity and Disattention, Problematic Relationships with Peers, and Prosocial Behaviors. The items are evaluated on a three-point Likert scale (i.e., Not True, Partially True, and Absolutely True). Cronbach’s α values ranged from 0.69 for Conduct problems to 0.85 for Hyperactivity–inattention; with average α equal to 0.75.

#### Academic Achievement

Given the variability in teacher grading behavior, to evaluate academic achievement we asked each teacher to examine the class grade records for the first semester and answer the following question *What was the child’s mean grade during the first semester?* Possible answers were on a three point Likert scale, with 1 meaning a failing grade (i.e., less than required for passing), 2 meaning an average grade, and 3 meaning a good or excellent grade.

#### Ethical Considerations

The University of Turin IRB approved the study (protocol no. 256088). School principals gave their consent for the students to participate in our study. The participants and their parents were presented with written consent request forms, describing the nature and objective of the study in compliance with the ethical code of the Italian Association for Psychology (AIP). We obtained signed informed consent from 89% students’ families, leading to a final number of 1940 students (54% males). The forms stated that data confidentiality would be assured and that participation in the study was voluntary. No incentives for participation were provided.

### Data Analysis

#### Clustering Techniques

For the analyses reported in this paper, and in order to overcome the problems linked to hierarchical cluster analysis, a two-step clustering procedure was followed: first, we ran a hierarchical analysis, using the Ward clustering algorithm, which is the most widely used algorithm in the Psychological and Social Sciences. Second, on the basis of the results of the first analysis (i.e., group centroids), we ran a second clustering procedure using the K-Means Procedure. This twofold approach is quite common in literature (e.g., see DiStefano et al., 2010) because it sums the benefits of both clustering approaches (for details, see Hair et al., 2006). We used the Squared Euclidean Distance as a measure of similarity. The number of clusters was decided according to three criteria: (a) the rescaled distances from the cluster dendrograms; (b) the amount of change in agglomeration coefficients at each step of the cluster analysis, and (c) the interpretability of the cluster solution (Hair et al., 2006).

#### Internal and External Validation

In order to validate the cluster solutions, given the adequate sample size, our sample was split randomly in two subsamples (i.e., a Development Sample and a Replication Sample). The analyses were initially performed on the Development Subsample, and then repeated on the Replication Subsample. Comparing the final results from the two sets of analyses allowed us to determine whether the final cluster analysis solution was an artifact or if it was capable of effectively representing groups underlying the population. As a final step the cross-validated cluster solution was applied to the whole sample.

In order to externally validate the typology, on the basis of the optimal cluster solution uncovered in the previous step of the analysis, differences among the subgroups were studied with regards to the following variables: gender, academic achievement, commitment, and student–teacher relationship (as assessed by the teacher).

#### Relationships Between Student Types and Their Behavior

In order to study the relationship between the clusters derived from the previous analyses and the behaviors exhibited by children in their classrooms, we ran a multivariate analysis of variance (MANOVA), with the SDQ subscales as dependent variables and the typology serving as the factor. Following this analysis, we ran univariate ANOVAs to study the differences amongst the clusters, where significant.

## Results

### Cluster Analysis

The clustering procedure described in the “Materials and Methods” section allowed us to obtain six clusters that were each based on a different typology of student.

To examine the characteristics of the six clusters, we performed a MANOVA on the peer nomination variables used in the clustering procedure, with the obtained typology serving as the factor. As expected, a significant multivariate effect emerged [Wilks’s lambda = 0.019, *F*(40,1794) = 66.72, *p* < 0.001, $\eta_{p}^{2}$ = 0.548), indicating that about 55% of the variability in student nominations was accounted for by group differences amongst the six clusters. **Table 1** presents descriptive statistics (i.e., *M* and *SD*), univariate ANOVA tests of significance, pairwise comparisons, and effect sizes for the analyses. As can be seen in **Table 1**, all ANOVAs were significant.

**Table 1:** Cluster characteristics: M (SD), univariate ANOVA tests, pairwise comparisons (Bonferroni), and partial eta-squared.

	Student type means								
Peer	Cluster 1 –	Cluster 2 –	Cluster 3 –	Cluster 4 –	Cluster 5 –	Cluster 6 –	*F*(5,418)	*p*	η^2^
nomination	Very liked/	Liked/Average	Less liked/	Low prosocial/	Less liked/	Very liked/			
variable	Prosocial	Prosocial	Disturbing	Non-disturbing	Non prosocial	Disturbing			
	(*n* = 52, 12%)	(*n* = 111, 26%)	*(n* = 41, 10%)	(*n* = 142, 34%)	(*n* = 59, 14%)	(*n* = 19, 5%)			
Playing	39.35 (14.91)ˆa	28.75 (10.08)ˆb	14.27 (12.43)ˆc	14.28 (9.09)ˆc	13.22 (8.84)ˆc	45.46 (9.96)ˆd	83.386	<0.001	0.499
Doing classwork	40.28 (15.61)ˆa	20.97 (9.59)ˆb	10.25 (8.61)ˆc	10.53 (7.92)ˆc	8.09 (6.75)ˆc	35.45 (15.01)ˆa	99.256	<0.001	0.543
Disturbing	3.53 (6.29)ˆa	7.64 (9.39)ˆb	82.94 (10.38)ˆc	7.34 (8.34)ˆb	42.62 (20.12)ˆd	49.17 (22.78)ˆe	373.739	<0.001	0.817
Helping	49.76 (14.09)ˆa	28.92 (12.61)ˆb	11.37 (11.86)ˆc	10.14 (7.20)ˆc	8.25 (8.30)ˆc	30.57 (16.61)ˆb	138.125	<0.001	0.623
Arguing	6.64 (8.95)ˆa	5.84 (7.39)ˆa	62.79 (18.71)ˆb	7.28 (8.99)ˆa	29.40 (13.62)ˆc	41.25 (15.12)ˆd	230.697	<0.001	0.734
Being isolated	6.64 (10.26)ˆa	7.04 (10.63)ˆa	27.41 (19.61)ˆb	5.45 (6.49)ˆa	21.12 (21.30)ˆc	10.41 (7.40)ˆa	30.320	<0.001	0.266
Getting along with teachers	56.21 (17.07)ˆa	20.89 (13.75)ˆb	10.82 (10.52)ˆc	10.28 (9.99)ˆc	12.06 (10.31)ˆc	25.99 (14.04)ˆb	117.106	<0.001	0.583
Getting laughed at	12.12 (11.95)ˆa	8.10 (8.82)ˆb	32.41 (19.15)ˆc	8.01 (7.17)ˆb	26.14 (17.23)ˆd	17.57 (12.07)ˆa	46.379	<0.001	0.357

*Column entries with different superscripts differ from each other at least at p < 0.01.*

### Internal Validation

In order to internally validate the obtained typology, a five-step cross-validation procedure was employed: (1) Data were divided randomly into two samples; (2) A cluster analysis was performed on the first sample; (3) A cluster analysis was run on the second sample; (4) The second sample was classified into clusters by a K-Means cluster analysis, in accordance with the cluster centroids derived from the first subsample; (5) The agreement was computed between the two classifications obtained on the second sample. Cohen’s κ was used to determine the level of agreement between the two cluster solutions. The agreement between cluster solutions was very good: κ = 0.827 (95% CI, 0.770 to 0.884), *p* < 0.001, confirming the stability of our classification.

**FIGURE 1:**
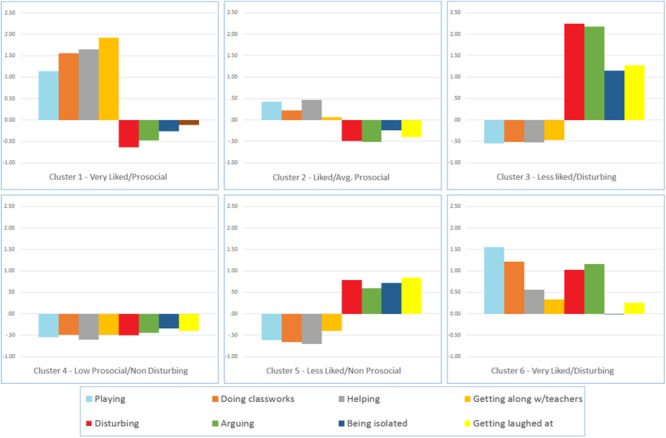
Cluster mean values for standardized peer nomination variables. Cluster 1 (*n* = 52, 12.3%), cluster 2 (*n* = 111, 26.2%), cluster 3 (*n* = 41, 9.7%), cluster 4 (*n* = 142, 33.5%), cluster 5 (*n* = 59, 13.9%), and cluster 6 (*n* = 19, 4.5%).

**Figure 1** depicts the final cluster profiles for the whole sample, expressed in standardized scores.

### Clusters Description

Cluster 1, *Very liked children with prosocial behaviors*, comprises around 12% of the sample. Children in this group exhibit high scores in the dimensions “Playing,” “Doing classwork,” the highest scores in “Helping” and “Getting along well with the teacher,” and low scores in all other dimensions.

Cluster 2, *Liked children with average prosocial behaviors*, represents 26% of the sample. Children in this group exhibit medium scores in the dimensions “Playing,” “Doing classwork,” “Helping,” and “Getting along well with the teacher,” but without reaching the selected percentages that characterize Clusters 1 and 6. Children in this group also obtain medium-low or low scores in the other dimensions.

Cluster 3, *Less liked children with disturbing behaviors*, covers 10% of the sample. Children in this group have obtained the highest scores in the following dimensions: “Disturbing,” “Arguing,” “Being isolated,” “Getting laughed at” and very low scores in all the other dimensions.

Cluster 4, *Children with low prosocial but non-disturbing behaviors*, is the most numerous cluster and corresponds to around 34% of the sample. Children in this group have low scores in all dimensions.

Cluster 5, *Less liked children with non-prosocial behaviors*, is made up of 14% of the total sample. Children in this group receive the lowest number of peer choices and exhibit medium-high scores in the following dimensions: “Disturbing,” “Arguing,” “Being isolated,” “Getting laughed at,” and low scores in the other behavioral dimensions.

Cluster 6, *Very Liked children with disturbing behaviors* is made up of 5% of the total sample. These children exhibit high scores as “Playing” and ”Helping,” and high scores in the items “Disturbing” and “Arguing.”

### External Validation

For gender and academic achievement, Chi-Square tests were performed to examine the relationship of these two variables with the identified typology. Both tests were significant, indicating the presence of a meaningful association (see **Table 2**).

**Table 2:** Clusters by gender and academic achievement.

	Student type distribution							
		Cluster 1 –	Cluster 2 –	Cluster 3 –	Cluster 4 –	Cluster 5 –	Cluster 6 –	*Chi-*	*p*
		Very liked/	Liked/	Less liked/	Low prosocial/	Less liked/	Very liked/	*squared*	
		Prosocial	Average prosocial	Disturbing	Non-disturbing	Non-prosocial	Disturbing		
Gender	Male	28.8%	53.2%	95.1%	52.1%	74.6%	84.2%	56.53	<0.001
	Female	71.2%	46.8%	4.9%	47.9%	25.4%	15.8%		
Achievement	Low	8.3%	22.0%	48.1%	30.0%	29.0%	17.6%	57.29	<0.001
	Average	11.1%	36.0%	48.1%	40.0%	58.1%	29.4%		
	High	80.6%	42.0%	3.7%	30.0%	12.9%	52.9%		

Concerning gender, Clusters 2 and 4 were quite balanced, while most of the students in the 3, 5, and 6 Clusters were males, and there was a majority of females in Cluster 1.

As regards achievement, the cluster with the maximum percentage of high achievement was Cluster 1, followed by Clusters 6, 2, 4, 5, and 3, which was the cluster with the lowest percentage of students with high achievement, and the highest percentage of low achievers. Other than Cluster 3, the highest percentages of students with low achievement were found in Clusters 4 and 5.

With respect to the Student–Teacher relationship, all the STRS subscales presented significantly different means across the clusters (see **Table 3**). The most relevant effect of the typology emerged for the Conflict subscale: the highest mean was found for Cluster 3, followed by Clusters 5 and 6. Clusters 1, 2, and 4 showed lower levels of Conflict. For the Closeness and Dependency subscales, the differences that were found were less relevant. Cluster 3 showed the highest level of Dependency, even if it was not significantly different from Cluster 5.

**Table 3:** Typology and STRS subscales: Descriptive statistics, univariate ANOVA tests, pairwise comparisons and partial eta-squared.

	Student type means								
STRS	Cluster 1 –	Cluster 2 –	Cluster 3 –	Cluster 4 –	Cluster 5 –	Cluster 6 –	*F*(5,418)	*p*	η^2^
subscales	Very liked/	Liked/	Less liked/	Low prosocial/	Less liked/	Very liked/			
	Prosocial	Average prosocial	Disturbing	Non-disturbing	Non-prosocial	Disturbing			
Closeness	33.42 (5.73)ˆa	31.45 (5.90)ˆa,b	27.15 (6.49)ˆc	30.73 (6.65)ˆa,b,d	28.68 (6.43)ˆc,d	28.00 (6.57)ˆb,c,d	6.527	<0.001	0.074
Conflict	11.02 (1.67)ˆa	13.35 (4.88)ˆa,b	26.93 (8.99)ˆc	14.02 (5.63)ˆb	20.05 (8.92)ˆd	19.42 (9.99)ˆd	42.593	<0.001	0.341
Dependency	5.37 (2.35)ˆa	6.09 (2.66)ˆa,b	9.17 (4.22)ˆc	6.70 (3.03)ˆb,d	7.60 (3.41)ˆc,d	6.21 (1.96)ˆa,d	9.445	<0.001	0.103

*Column entries with different superscripts differ from each other at least at p < 0.01.*

### Relationships Between Student Types and Behaviors

The MANOVA conducted on the SDQ subscales, using the typology as a predictor, showed a significant multivariate effect [Wilks’s lambda = 0.047, *F*(25,1487) = 13.24, *p* < 0.001, $\eta_{p}^{2}$ = 0.139]. As shown in **Table 4**, univariate ANOVA tests were all significant.

**Table 4:** Effects of typology on SDQ subscales: Descriptive statistics, univariate ANOVA tests, pairwise comparisons and partial eta squared.

	Student type means								
SDQ subscales	Cluster 1 –	Cluster 2 –	Cluster 3 –	Cluster 4 –	Cluster 5 –	Cluster 6 –	*F*(5,418)	*p*	η^2^
	Very liked/	Liked/	Less liked/	Low prosocial/	Less liked/	Very liked/			
	Prosocial	Average prosocial	Disturbing	Non-disturbing	Non-prosocial	Disturbing			
Emotional problems	1.13 (1.37)ˆa	1.92 (2.07)ˆa,b	3.73 (2.46)ˆc	2.22 (2.13)ˆb,d	3.05 (2.41)ˆc,d	2.37 (2.34)ˆa,d	8.815	<0.001	0.098
Behavioral problems	0.48 (1.06)ˆa	1.04 (1.56)ˆa,b	5.25 (2.25)ˆc	1.48 (1.75)ˆb,d	3.65 (2.36)ˆe	2.42 (1.77)ˆd,e	52.040	<0.001	0.392
Hyperactivity problems	0.87 (1.5)ˆa	2.54 (2.85)ˆb	7.83 (2.25)ˆc	3.12 (2.59)ˆb	5.65 (2.58)ˆd	5.84 (3.17)ˆc,d	48.718	<0.001	0.376
Peer problems	0.94 (1.26)ˆa	1.43 (1.69)ˆa	4.2 (2.27)ˆb	1.67 (1.92)ˆa	3.76 (2.64)ˆb	1.58 (2.04)ˆa	25.020	<0.001	0.236
Prosocial behavior	8.73 (1.55)ˆa	7.3 (2.33)ˆb	4.08 (2.21)ˆc	6.97 (1.99)ˆb	5.33 (2.45)ˆc,d	6.26 (1.85)ˆb,d	28.818	<0.001	0.263

*Column entries with different superscripts differ from each other at least at p < 0.01.*

Concerning problematic behaviors, Cluster 3 showed the highest levels for all the subscales: Emotional problems, Behavioral problems, Peer problems, and Hyperactivity problems. Cluster 3 has also the lowest level of Prosocial Behavior. Cluster 5 shows a similar trend as Cluster 3, with levels of Emotional and Peer problems, and of Prosocial behavior that were not significantly different. Cluster 1 has the lowest levels of problems and the highest level of Prosocial behavior.

## Discussion

The primary aim of our study was to identify and describe the characteristics of different socio-behavioral patterns in children attending the first grades of primary school. The novelty of the study was to base the development of the typology on two elements: popularity among peers and peer perception of schoolmates’ behaviors. We used cluster analysis to investigate these patterns, and we identified six different categories of students. The most frequent behavioral pattern belonged to Cluster 4, *Children with low prosocial but non-disturbing behaviors*. These children did not have leadership roles in their classroom, but they were not completely isolated either. Their characteristics for peer group insertion were, therefore, similar to the description of children with low social impact given by Coie et al. (1982). The second most numerous behavioral pattern was Cluster 2, *Liked children with average prosocial behaviors*. The children that belonged to this cluster presented similar characteristics to those of children in Cluster 4 (i.e., *Children with low prosocial but non-disturbing behaviors*). However, children in Cluster 2 differentiated themselves because they exhibited a higher level of social preference, granted to them by their peers, as well as a higher amount of prosocial behaviors.

The remaining clusters were decidedly less numerous. Cluster 5 (*Less liked children with non-prosocial behaviors*), contained children characterized by low scores in social preference. Furthermore, these children obtained numerous nominations in the items that investigated antisocial and asocial behaviors, and few nominations for prosocial behaviors. Antisocial and asocial behaviors are even more present in the children belonging to Cluster 3 (*Less liked children with disturbing behaviors*) who presented slightly higher levels of social preference compared to those of the children from Cluster 5. These two clusters overlap in terms of low levels of social preference, but they differ on the basis of co-occurring behaviors. Cluster 3, is connected to those children that were labeled as “Troubled” in previous studies (e.g., Estell et al., 2008; Robertson et al., 2010). By observing the characteristics of Clusters 3, 4, and 5, it can be noticed that low levels of social preference co-occur with low levels of prosocial behaviors which, in turn, are not necessarily associated with the presence of high levels of disturbing and contentious behaviors.

As we had speculated previously, we observed a behavioral pattern that distinguished itself for the presence of high levels in both popularity and prosocial behaviors, combined with the absence of antisocial and asocial behaviors. Children from Cluster 1 (*Very liked children with prosocial behaviors*) shared the characteristics of the popular children identified through sociometric measures (Coie et al., 1982).

In closing, and in agreement with our hypotheses, we registered the presence of a cluster (Cluster 6, *Very liked children with disturbing behaviors*) that was the least numerous for size, and that was characterized by high levels of antisocial behaviors, combined with good levels of social acceptance and prosocial behaviors. Students belonging to this cluster have peculiar traits that can be read in the light of resource control theory (Hawley, 2008). This cluster, in fact, distinguishes itself because it is formed by a group of children that are chosen by their peers for both play activity and schoolwork, all the while presenting disrupting and contentious behaviors. Therefore, the presence of said behaviors does not seem to have an effect on their social pleasantness as perceived by their peers. It appears that Disruptive and Contentious behaviors do not necessarily lead to peer group isolation but, instead, can be associated with good levels of social acceptance. Said behaviors, along with other characteristics of these children’s behaviors, are not necessarily maladaptive, but they can be used for maintaining good relations with their classmates (Farmer et al., 2003; Roseth et al., 2011).

Concerning the quality of the student–teacher relationships, teachers perceive more conflict in the relationships with children who are characterized by disturbing and contentious behaviors (Clusters 3, 5, and 6) as previously highlighted by Hamre et al. (2008). Our results, through the evaluation of the teacher’s perception of the relationship, allow us to highlight an additional aspect of the classroom relationships that characterize the children in Cluster 6. In fact, teachers perceive these relationships to be characterized by high levels of Conflict and low levels of Closeness, and they do not seem to notice all the behavioral variations and co-occurrences that emerge from our study. Therefore, the analysis that we have conducted might prove to be a useful tool for teachers because it would grant them a better knowledge of their class, as well as give them the chance to reflect on the link between a pupil’s association to a specific cluster and their own perception of their relationship with said pupil.

Considering the pupils’ academic performance, the results confirm the data in the literature that highlight the association between poor peer group relations and low academic performance (e.g., Rubin et al., 1998).

With regards to gender, it was distributed differently throughout the various clusters. In fact, females were more present in the clusters that were characterized by elevated levels of social preference combined with prosocial behaviors (Cluster 1) and males were more present in the clusters characterized by disruptive behaviors (Clusters 3, 5, and 6). In particular, there was a higher presence of males in Cluster 6, characterized by the co-occurrence of prosocial behaviors with high levels of social preference, and disturbing and contentious behaviors. The findings are in line with what has been highlighted by resource control theory.

The final aim of our study was to investigate cluster predictivity with reference to some characteristics of the child’s behavior, as observed by the teacher. Cluster membership was a good predictor of some aspects of the child’s social behavior as perceived by teachers. In fact, the results confirmed that a lower presence of social weaknesses and high levels of prosociality characterize children who belong to Cluster 1(*Very liked children with prosocial behaviors*), whereas children in Cluster 3 (*Less liked children with disturbing behaviors*) present significantly higher levels of Emotional Symptoms, Behavioral Problems, Hyperactivity and Lack of Attention, Problematic Relationships with Peers and lower levels of Prosocial Behaviors. It should be noted that, also in this case, the characteristics of children that belong to Cluster 6 (*Very liked children with disturbing behaviors*) are in line with what has emerged from peer nomination concerning disturbing and contentious behaviors: these children have rather high scores in the scales that are relative to weak points, in particular on the scale that concerns Hyperactivity and Lack of Attention.

## Conclusion and Theoretical Implications

As a final remark, we believe that our study contributes significantly to the literature because it enlarges the present considerations on perception discrepancies between students and teachers (Pepler and Craig, 1998; Pellegrini and Bartini, 2000). In fact, teachers do not notice certain behavioral characteristics that are instead reported by children when they distinguish clusters among their peers. For example, Cluster 4 is characterized by a low presence of prosocial behaviors, but this aspect has not been noticed by teachers. Furthermore, teachers notice the high levels of hyperactivity and lack of attention that are present in the children from Cluster 6, but seem to ignore their prosocial characteristics, which are instead pointed out by their peers. Therefore, we can deduct the importance of basing studies on the peer group’s point of view, especially to make up for the teachers’ difficulties in recognizing prosocial abilities in children who present externalizing behaviors (Bouchard et al., 2006). We can also recognize an additional discrepancy between peer group perception and the teacher’s perception for what concerns the quality of the student–teacher relationship of the children from Cluster 6. On one side, teachers perceive a high level of conflict with these children. On the other side, peers report that these children get along well with their teachers and that the teachers enjoy spending quite a lot of time with them. This discrepancy in perception could be owed to the different meaning given by peers and teachers to the amount of time that the latter spend with children from Cluster 6: peers see it as a sign of the attention and care that is present in the relationship, while teachers see it as a hard, inefficient task, characterized by elevated demands of energy.

### Implications for Educational Policy and Practice

The present study has relevant implications for those who deal with research and/or education in school settings, such as teachers, and school-psychologists. The clusters that we have identified allowed us to grasp the complexity of the classroom, and could give teachers and school psychologists a useful framework through which to observe social skills profiles. Said interpretative framework is ever more useful the more it is precocious, seeing as the first years of schooling are very important for the child’s development (Pianta et al., 1995). This knowledge can help teachers and school psychologists to plan early interventions, aimed at increasing the single pupil’s odds of having a good emotional and social development (Dymnicki et al., 2011; Bulotsky-Shearer et al., 2012). Furthermore, given that the source of information for this typology is the peer group, both school psychologists, and teachers with low level of familiarity with the classroom (e.g., new teachers or substitutes) could use the approach we have proposed to rapidly gain the knowledge about the classroom that is needed to adjust their interventions to the specific context.

In fact, the possibility of describing each cluster’s specificity, could help, if confirmed by other studies, to adapt educational strategies to students’ individual needs. For example, the traditional viewpoint of variable-centered studies has highlighted the need for specific interventions in the presence of aggressive behaviors, which were considered maladaptive (Hawley et al., 2007). Our results, in line with the studies based on resource control theory, allow us to grasp the dual and opposite social value of disruptive behaviors, with or without peer group acceptance. Operators in scholastic settings can now grasp the “social potential” of children who present aggressive behaviors while preserving good social standing (Rodkin and Roisman, 2010).

Having used the peers’ point of view when creating the clusters is a strong point in our study, because it can allow experts to grasp the nuances of some behaviors that might be hard to spot otherwise, or might be influenced by their personal traits (Lancelotta and Vaughn, 1989; Hamre et al., 2008). Cluster 6 is an example of this kind of discrepancy: the children’s peers, when choosing, highlight the social skills possessed by those who belong to this group, although they recognize the disturbing behavioral elements. For the teachers, instead, these behaviors are associated with conflictual relationships, and they are characterized by low levels of Closeness. On the other hand, becoming aware of the position that these children occupy in their peer group can help teachers recognize and give value to their positive traits. This can both modify teachers’ representations and expectations of students who are *Very Liked Children with disturbing behaviors*, reducing the Rosenthal effect, and it can also influence these children’s perception of themselves in a positive manner. Finally, this new awareness could presumably lead to a lowering of these children’s disturbing and contentious behaviors. The long-term effects are much more meaningful in the Italian context, because it has made the continuative presence of a teacher in an elementary school class group one of its cardinal principles.

### Limitations and Perspectives

Our study suffers from some limitations. First of all, the sample that we have considered is not representative for the Italian student population. For this reason, a replication study with a representative sample is needed. Furthermore, in order to study the generalizability of the findings it would be appropriate to replicate the study in different cultural settings.

We are currently planning a longitudinal study aimed at investigating whether cluster configuration may vary in different school grades, in light of the different meaning that behaviors acquire inside them (Pellegrini, 2008; Roseth et al., 2011). Similarly, it would be interesting to analyze the influence of schools (e.g., district, type of users), teachers, and the class-group on behaviors. For what concerns the class-group’s characteristics, it would be particularly interesting to investigate whether different compositions in the class, in terms of gender distribution, might influence the presence and distribution of the various clusters themselves.

## Author Contributions

CL, LP, and TP were involved with the design and interpretation of this work as well as writing and revising the manuscript. LP and TP were involved in the acquisition of the data. MS and DM analyzed the data and contributed to the revising of the manuscript.

## Conflict of Interest Statement

The authors declare that the research was conducted in the absence of any commercial or financial relationships that could be construed as a potential conflict of interest.
